# Complicated Crown Fracture of Permanent Incisors: A Conservative Treatment Case Report and a Narrative Review

**DOI:** 10.3390/bioengineering9090481

**Published:** 2022-09-18

**Authors:** Mateusz Radwanski, Corrado Caporossi, Monika Lukomska-Szymanska, Arlinda Luzi, Salvatore Sauro

**Affiliations:** 1 Department of Endodontics Chair, Conservative Dentistry, Endodontics Medical University of Lodz, 251 Pomorska Str., 92-213 Lodz, Poland; 2 Independent Researcher, Roma, 65B, 00030 Labico, RM, Italy; 3 Department of General Dentistry, Medical University of Lodz, 251 Pomorska Str., 92-213 Lodz, Poland; 4 Group of Dental Biomaterials and Minimally Invasive Dentistry, Department of Dentistry, Cardenal Herrera-CEU Universities, C/Santiago Ramón y Cajal, s/n., Alfara del Patriarca, 46115 Valencia, Spain; 5 Department of Therapeutic Dentistry, I. M. Sechenov First Moscow State Medical University, 119146 Moscow, Russia

**Keywords:** traumatic dental injury, tooth fracture, adhesive reattachment

## Abstract

Dental trauma may have a severe impact on the social and psychological wellbeing of a patient. Most cases of dental injuries involve anterior teeth, especially the maxillary upper incisors. Crown fractures, with or without pulp exposure, are the most common trauma in permanent dentition. There are many methods of management, in which the initial state of the pulp, the time since the injury, and the presence of an accompanying injury play a key role. This case report aimed at showing a possible conservative treatment after complicated tooth fracture that consisted of partial pulpotomy followed by adhesive reattachment of the tooth fragment using a technique based on heated resin composite. Such a specific procedure represents a conservative approach to traumatic coronal lesions, providing a suitable opportunity to maintain the tooth vitality, aesthetics, and function. Indeed, reattachment of tooth fragment using a composite/adhesive is a simple technique to achieve excellent results in terms of aesthetic and function.

## 1. Introduction

Traumatic dental injuries (TDIs) concern mostly children and young adults [1]. TDIs may have a severe impact on the social and psychological wellbeing of a patient. Dental fractures are classified according to the fractured tissue and pulp involvement, and include enamel infractions, uncomplicated crown fractures (enamel fractures and enamel–dentin fractures), complicated crown fractures (enamel–dentin fractures with pulp exposure), and crown–root and root fractures [2]. Most of the cases of dental injuries involve anterior teeth, especially the maxillary upper incisors [3,4]. Conversely, lower central incisors and upper lateral incisors are less commonly involved [5,6]. Crown fractures, with or without pulp exposure, are the most common type of trauma in permanent dentition [3,7]. Complicated crown fractures involve enamel and dentin with pulp exposure; the occurrence of such trauma ranges between 2% and 13%, and the most common causes are falls, traffic accidents, domestic violence, fights, and sports [3,7].

The treatment of complicated crown fractures according to the International Association of Dental Traumatology (IADT, 2020) [1] includes conservative treatment of the pulp, such as partial pulpotomy, in both mature and immature roots. It is worth emphasizing that the condition of the pulp before starting treatment should be determined [1]. However, in the case of tooth injuries, it is possible to obtain false negative results, meaning no response despite vital pulp. After the trauma, the nervous response is temporarily lost, so the sensibility test (cold test and electric pulp test) may be negative [8]. Nevertheless, it is recommended that pulp vitality testing is performed after injury, as well as at follow-up visits, to assess any possible change over time [1,9]. In addition, tests for the evaluation of blood flow in the pulp, such as laser Doppler flowmetry (LDF), should be used [1].

During diagnosis, it is strongly recommended to take a parallel periapical radiograph. Additional radiographs are required if there are signs and symptoms of other potential injuries. For soft tissue injuries, X-rays of the lip and/or cheek are needed to look for tooth fragments or external debris. In the event of suspicion of other injuries, especially root fractures, crown–root fractures, or lateral luxations, the clinician should consider using cone beam computerized tomography (CBCT). This examination enables the determination of the location, extent, and direction of the injury [10]. The decision on additional patient exposure to radiation should be based on the fact that the obtained results will change the route of injury management.

When a tooth fragment is available, it should be reattached; if it is not available, it is recommended to cover the dentin with a glass-ionomer or a bonding agent and composite resin [1]. If a post is required to retain a crown in a mature tooth with complete root formation, root canal treatment is the preferred method.

The favorable outcomes include asymptomatic teeth with positive response to pulp sensibility testing, good quality restoration, and continued root development in immature teeth. Treatment outcome depends on the severity of the injury, quality, and timeliness of initial care, and recall protocol (after 14 days, 6–8 weeks, 3 and 6 months, and one year after the injury) [1]. The latter was performed via radiographic assessment and cold/hot vitality test. The present case report aimed at showing a possible conservative treatment after complicated tooth fracture that consisted of partial pulpotomy followed by adhesive reattachment of the tooth fragment.

## 2. Presentation of Cases

### 2.1. Diagnosis

**Figure 1:**
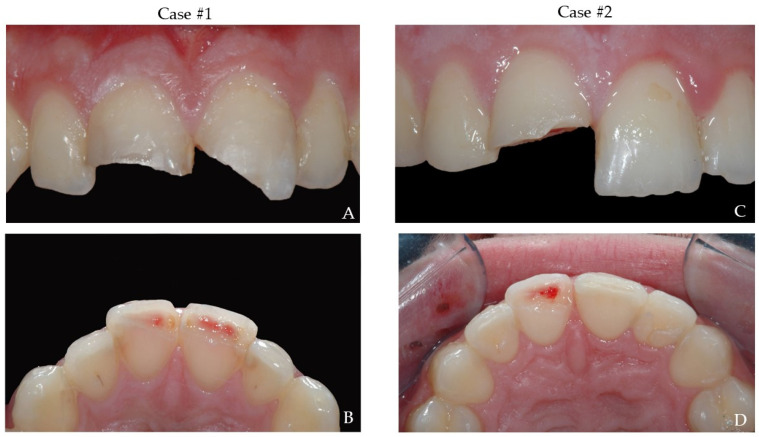
Intraoral photographs of both cases. (**A**,**C**)—buccal view; (**B**,**D**)—occlusal view.

A 15 year old male patient (case #1) experienced a blunt trauma during basketball game. The patient suffered from a complicated fracture of the crowns of 11 and 21 (Figure 1A,B). The fragments were recovered and kept immersed in milk until the appointment two days after the trauma.

In case #2, a 21 year old male patient reported to dental office immediately after a complicated crown fracture of the crowns of 11 during a tennis game (Figure 1C,D). The dental fragment was immediately stored in Hank’s balanced saline solution.

**Figure 2:**
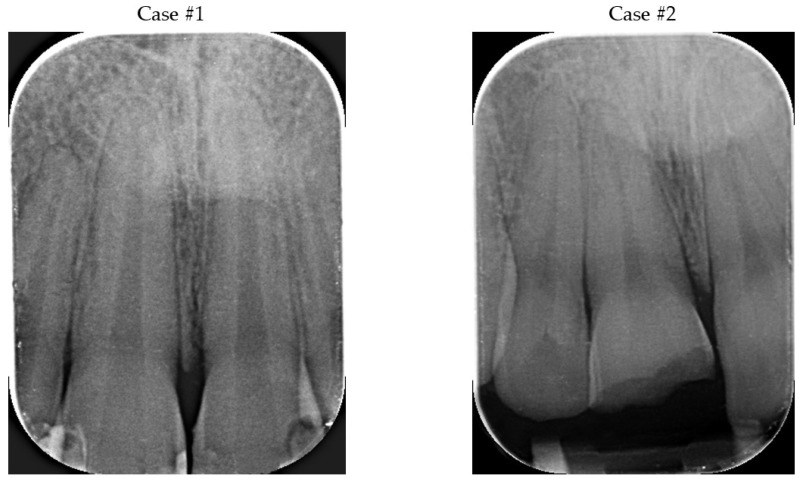
The preoperative X-rays.

**Figure 3:**
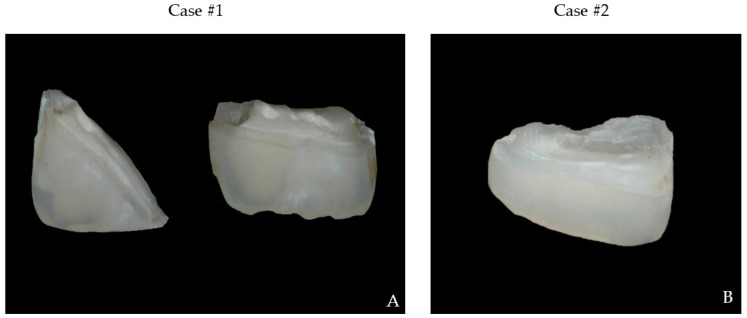
Fragments of teeth (**A**) 11 and 21, (**B**) 11.

In both cases, teeth were vital with thermal and electrical stimulus; no mobility and symptoms of other trauma were detected during clinical and X-ray examination (Figure 2A,B). The tooth fragments were not damaged, fit the tooth crowns, and did not interfere with the occlusion (Figure 3A,B).

### 2.2. Pulp Treatment Option

In case of complicated crown fractures, vital pulp therapy (VPT) interventions include direct pulp capping (DPC), partial pulpotomy (PP), and complete pulpotomy (CP) (Table 1).

Based on the analysis of the clinical conditions and additional tests, it was decided to perform PP with adhesive/composite reattachment in case #1, as well as in case #2, maintaining the vitality of the treated teeth. PP was chosen as the treatment method following the admission of patients within 14 days after injury, no caries, and vital and asymptomatic pulp.

In general, VPT has a high success rate. However, clinical factors such as the vitality of the pulp, the time from exposure to intervention, age of the patient, other coexisting injuries, the cause of exposure, and the extent of exposure may influence the favorable outcome [11]. Additionally, PP has the advantage of preserving the cell-rich coronal pulp, which may provide greater healing potential, and so maintain the possibility of having physiological dentin deposition [12].

The diagnosis of injured tissue plays a key role, which can often be difficult in the case of trauma. Additionally, the tests used for sensitivity assessment (electric and/or thermal stimulation), which are dependent on neural response, may not be reliable in the first days after trauma [13]. In the case of difficulties, tests based on the measurement of actual blood flow, such as LDF or pulse oximetry, are recommended [13]. The increasing time between injury and intervention contributes to lower treatment success [11,14]. The age of the patient, and, thus, root development, does not influence the treatment prognosis [11]. It was believed that the pulp of the elderly contains more fibrous than cellular elements, which means that it has less repair capacity. Currently, it is thought that more important factor for treatment success is the complete removal of infected tissue, and, therefore, biological methods can be successfully applied to elderly individuals [15]. However, the presence of a further injury (e.g., luxation) associated with the crown fracture may adversely affect the outcome of the treatment [16]. The pulp exposed due to trauma, compared to carious exposure, has a greater chance of healing [11,17]. The extent of pulp exposure does not affect treatment prognosis, provided that it remains vital [11].

It is important to consider that the success of VPT is also influenced by factors such as proper infection control (rubber dam isolation), bleeding control, selection of the capping material providing a tight seal, and a final restoration.

### 2.3. Preparation of Operatory Field

In the presented cases, after administrating the anesthesia of 2% lidocaine with 1: 80,000 adrenalines not alkalinized (Dentsply, Konstanz, Germany), and performing a total rubber dam isolation to avoid cross-contamination, the partial pulpotomy was executed through high-speed bur under continuous saline irrigation.

Various local anesthetics and different vasoconstrictors concentration are used in VPT after trauma. Based on the available literature, the active substance and its concentration demonstrated no effect on the treatment results [18]. On the other hand, the vasoconstrictors may affect hemostasis, thus, making it difficult to assess the initial state of the pulp [18]. However, there are no clear guidelines for vasoconstrictor application in vital therapy.

The ability to control bleeding is an important factor determining the success of treatment, as prolonged bleeding is supposed to be a sign of an irreversible inflammatory process. The time needed to stop bleeding varies from 2 to 25 min, with no effect on the prognosis of pulpotomy [18]. Moreover, achieving hemostasis at the site of exposure does not provide an accurate assessment of pulp inflammation at the level of canal orifice [19]. Interestingly, immediate hemostasis does not determine the success of VPT [20]. Additionally, high blood flow may indicate good blood supply to the pulp, and high level of ability to repair and maintain vitality [20,21].

For bleeding control, the hemostatic agents are recommended. True hemostatic agents such as ferric sulphate or hydrogen peroxide are not recommended, due to the risk of masking the inflammation of the radicular pulp [21]. Several hemostatic products are available i.e., sodium hypochlorite, chlorhexidine, lidocaine with a vasoconstrictor, or saline, that show no adverse reaction to a pulp [22]. According to the guidelines (2021) of the American Association of Endodontists (AAE), the use of sodium hypochlorite is recommended, due to its bactericidal properties, and the ability to remove fibrin, clot, biofilm, and discolorations [23]. The sodium hypochlorite can be used safely in direct contact with pulp tissue (passive irrigation or soaked cotton pellet) at various concentrations, without adverse effect to the pulp and treatment prognosis [23]. Thereby, to control bleeding, cotton pellets soaked with 1% sodium hypochlorite were applied and the hemostasis was obtained after 2 min in both cases.

### 2.4. Choice of Materials

An ideal pulpotomy material should be biocompatible, non-toxic, induce hard tissue formation, and exhibit a disinfecting properties [24]. The materials used in pulpotomy are calcium hydroxide (CH) and calcium silicate materials (CSMs) [24,25].

CH exhibits bactericidal properties, high pH, and, therefore, it is characterized by the ability to neutralize acids and lipopolysaccharides [26]. These preparations contribute to the dentin bridge formation and healing of the pulp [25]. The most recommended form is an aqueous suspension of CH, which is applied to cover both the exposed pulp and the adjacent dentin. Aqueous solutions cause fewer hydroxyl ions to be released, and are less toxic due to the low content of additives [25]. However, the light-cured materials based on CH are not recommended, due to the cytotoxicity of the monomer [27]. The major disadvantage of CH products is instability and resorption over time. Numerous studies show that the formed dentin bridge is heterogeneous, with the presence of pores (tunnel defects) that can be the entry points for microorganisms. In addition, the high pH of CH has a negative effect on the pulp, which may contribute to its extensive necrosis [28,29,30].

Most CSMs, such as mineral trioxide aggregate (MTA), are based on Portland cement, consist mainly of dicalcium or tricalcium silicates, and are mixed with water [31,32]. They are called “hydraulic” because they can set in contact with water. MTA stimulates the pulp cells to produce a dentine deposition (e.g., dentin bridge) [33]. During setting, calcium hydroxide is released, providing the antimicrobial properties. MTA, compared with CH, exhibits a higher mechanical strength, is less toxic, and causes less pulpal inflammation [34]. The disadvantages of MTA include the difficulty of application, long setting time, and teeth discoloration [35].

One of the major aesthetic complications after pulpotomy with white and grey MTA can be the discoloration of the tooth [36,37]. It is caused mainly by the oxidation of heavy metal oxides (bismuth or iron), and by the interaction between erythrocytes and the unset cement in case of inadequate hemostasis [38,39,40]. To prevent the discoloration, different approaches are suggested, such as using cements with alternative radiopacifying agents such as zirconium oxide, sealing the surrounding dentinal tubules with dentin bonding agents before MTA application, or adding zinc oxide or aluminium fluoride to the powder [41,42,43,44]. Unfortunately, there is no consensus regarding that matter and, consequently, no guidelines for the prevention of discoloration were issued.

**Figure 4:**
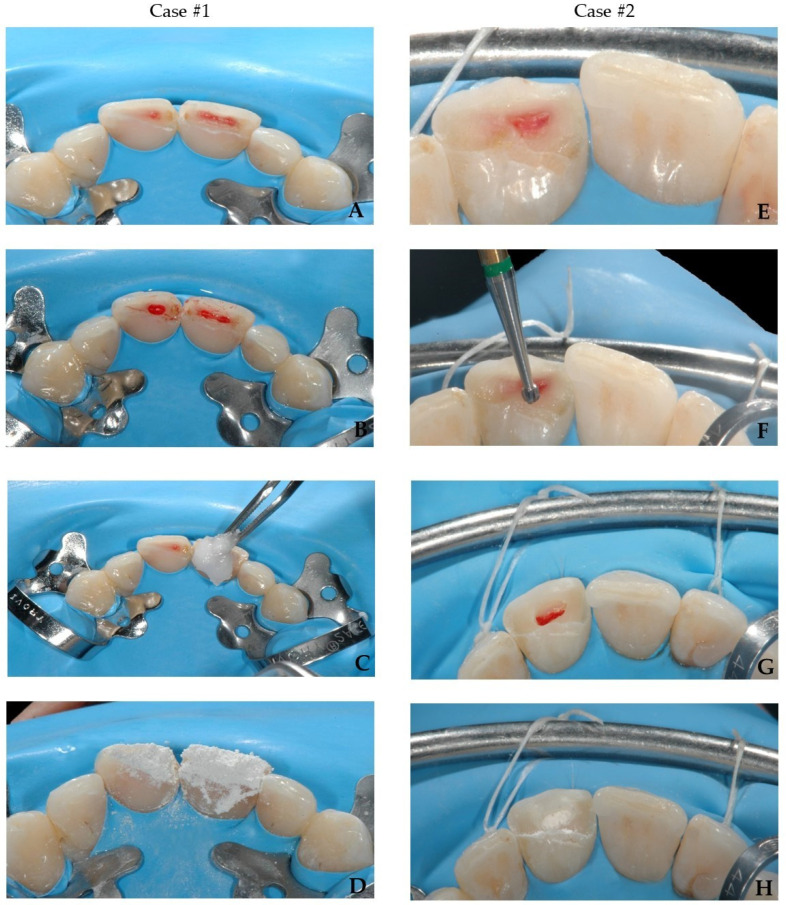
PP of teeth 11 and 21 (case #1) and PP of tooth 11 (case #2). (**A**,**E**)—clinical situation before pulpotomies; (**B**,**F**)—partial pulpotomies; (**C**,**G**)—hemostasis with cotton soaked with 1% sodium hypochlorite; (**D**,**H**)—capping with MTA.

The powder of MTA is mixed with distilled water (3:1 ratio) to obtain a wet, gel-like consistency [45]. For mixing, a metal or plastic spatula and a glass plate or paper can be used. The working time with the material is about 5 min, and the setting time is long, from 3 to 4 h. Some substances may affect the setting time, causing it to shorten (sodium hypochlorite) or elongate (saline, lignocaine) [45]. However, the results for chlorhexidine are contradictory; some studies show no effect [46], while others show an alteration in the setting of the MTA [47]. The excavator, a retrograde amalgam that carries, can be used for the delivery of the prepared MTA, and paper points, pluggers, or ultrasounds for the condensation. The material should not be condensed with excessive force, due to the risk of reducing its strength and surface hardness [48]. The material should be applied excessively to cover pulp and all walls without the risk of creating voids. For the final condensation, it is recommended to use a cotton pellet moistened with sterile water to initiate the setting reaction and clean the excess of the material. Appropriate material thickness in the case of pulpotomy amounts up to 3–4 mm. The thickness and compaction of the MTA layer can be assessed with X-ray [49]. The coronal excess of material should be removed with use of cotton pellet or mechanically with burs. In the presented cases, MTA (ENDOPASS, DEI -Italia, Varese, Italy) was mixed with bi-distilled water using a plastic spatula on a glass pad, and condensed with paper points and moist cotton pellet (Figure 4A–D); the excess of material was removed with cotton pellet.

Many systematic review papers and meta-analyses report that there is no significant differences between MTA and CH regarding the survival rate of pulp, both for permanent immature teeth and teeth with closed apices [12,50,51,52]. On the other hand, some studies indicate the superiority of MTA-based materials due to lower solubility when compared with CH, greater biocompatibility, and the quicker formation of thicker and more homogenous dentin bridge, which may be a greater barrier to bacterial leakage [53,54]. Therefore, in the presented cases, MTA was applied.

The calcific metamorphosis is an adverse effect of the MTA and CH application [51,52]. It is associated with the induction of odontoblasts to form hard tissues and can only take place when the pulp is vital. The incidence of pulp obliteration after dental trauma amounts from 4 up to 24% [55]. The calcification is not considered a criterion of success or failure of treatment, and only the obliteration of the pulp chamber may increase the difficulty of endodontic treatment in the future, and may favor perforation when trying to locate the canal orifice.

### 2.5. Restorative Procedure

When a well-hydrated intact tooth fragment is available, which fits to the remaining crown without interfering with the patient’s occlusion, the first-choice treatment should be adhesive reattachment of the fragment. It does not always have to be stored in a humid environment; a study shows that a dehydrated fragment was attached to the tooth with the use of additional retention elements, and after a 15 month follow-up, the tooth retained its vitality, functionality, and natural aesthetics [56]. However, the dehydration of the broken fragment may cause color disharmony with the tooth remnant, but such an issue disappears after about 12 months, due to the absorption of water by the fragment [57,58].

Compared to conventional restorative techniques, the reattachment of tooth fragments exhibits several advantages: the original shape, color, brightness, and texture of the enamel surface [59]. The incisal edges of reattached fragments tend to wear at a similar rate to adjacent natural teeth. This technique is less time-consuming, minimally invasive, and simple to perform [60]. The type of injury causing the crown fracture or the storage medium prior to reattachment can exert no effect on the survival, color, and bond strength of the treated teeth after reattaching the fragment [57]. It is very important to perform the reattachment procedure under a rubber dam to avoid contamination and deterioration of the adhesive layer [9].

Many different types of adhesive systems (total-etch, self-etch) and different intermediate materials e.g., paste and flowable composite materials, adhesives, or glass ionomer cements, can be applied [61,62,63,64,65]. There is no consensus in the literature regarding the ideal technique and material for reattachment of a tooth fragment [64].

Some authors recommend pre-reattachment/initial modification of remaining fragments, e.g., dentine grooves, over-contouring, chamfering, or beveling, to expand the enamel surface, increasing adhesion and ensuring higher fracture resistance of such restorations [66,67]. These additional retention elements should be considered if the broken fragment involves more than 50% of the clinical crown [68]. However, the most recent systemic review recommends simple reattachment without any modification to reduce the technical sensitivity of the procedure and the length of the clinical phase [64]. In addition, achieving imperceptible anterior restoration depends on operator clinical experience and knowledge of dental anatomy, rather than any previous preparation [69].

The bond strength between adhesive systems and calcium-silicate-based materials is also an important aspect. The in vitro results indicate that the bond strength of the resin-based materials to the MTA is favorable for the total-etch technique [70,71,72]. On the other hand, in the case of Biodentine, the bond strength when using self-etch or total-etch systems achieves similar results [73,74,75,76]. Due to the lack of evidence regarding the chemical interaction of self-etching materials with bioceramics, pre-treatment with phosphoric acid prior to the bonding procedure may be recommended [75].

**Figure 5:**
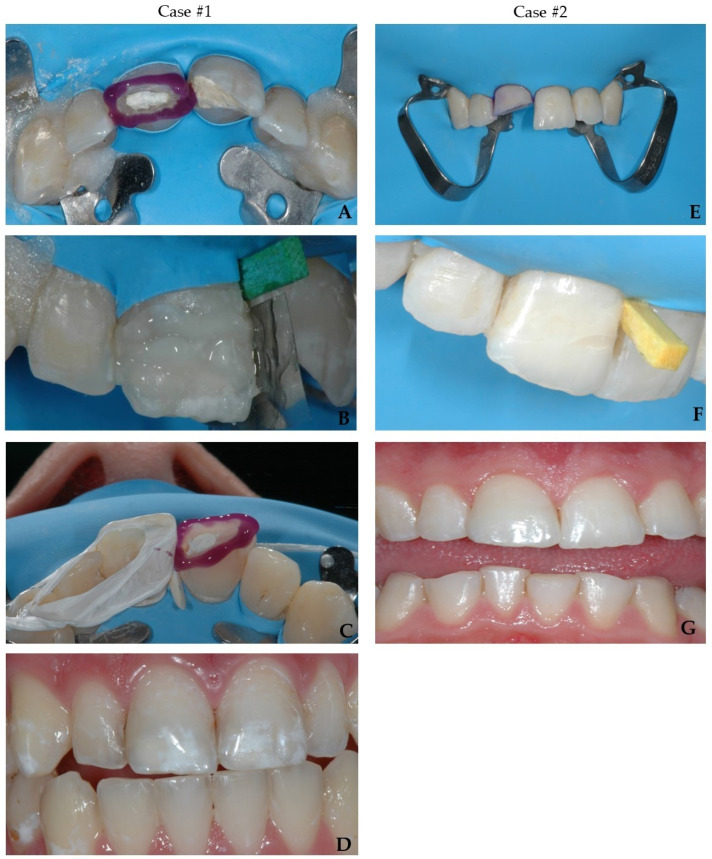
The reattachment of tooth 11 and 21 (case #1- up). (**A**)—selective etching of tooth 11; (**B**)—reattachment of coronal fragment of tooth 11; (**C**)—selective etching of tooth 21; (**D**)—buccal view after adhesive reattachment of teeth 11 and 21. The reattachment of tooth 11 (case #2- down). (**E**)—selective etching of tooth 11; (**F**)—reattachment of coronal fragment of tooth 11; (**G**)—buccal view after adhesive reattachment of tooth 11.

In the present cases, no modification was carried out before the reattachment procedure. In both cases, the selective enamel etching both on the tooth and the fragment was performed using a 36% phosphoric acid gel (DeTrey Conditioner 36, Dentsply Sirona GmbH, Bensheim, Germany) under rubber dam isolation. Then, a self-etching two-component adhesive system (Clearfil™ Se Bond, Kuraray Noritake Dental Inc., Tokyo, Japan, CSE) was applied as per manufacturer’s instruction, and air-dried with a strong stream of air for 5 s to completely remove the excess adhesive. This was finally light-cured for 20 s (Radii Xpert, Voco GmbH, Cuxhaven, Germany). Subsequently, a thin layer of enamel (Shade A2) mass composite (Asteria Tokuyama NE, Tokuyama Dental Corporation, Taitouku Tokyo, Japan) was applied directly on the tooth as an intermediate material to reattach the fragment to the tooth. The composite was heated up to 54 °C in a warming device (AdDent Calset™ Composite Warmer, AdDent Inc., Danbury, CT, USA) to increase the degree of polymerization, a better adaptation of the fragment on the tooth and to provide easier management of the excess removal [77]. Next, photopolymerization was carried out for 10 s. To avoid undesirable inhibition layer due to photopolymerization, the area was protected by covering the tooth with an oxygen inhibitor gel (Oxyguard II, Kuraray Europe GmbH, Frankfurt, Germany) and polymerized for 40 s. (Figure 5A–G).

### 2.6. Occlusion Adjustment

Occlusal adjustment involves the development of an acceptable central relation contact position for the patient, ensuring acceptable lateral and protrusion guidance. This is necessary to eliminate premature contacts and bad guidance, which could contribute to excessive forces concentrating within the reattached fragment, thus, causing it to detach. Therefore, the presented cases were performed using a diamond bur (#3118F—KG Sorensen, Cotia, SP, Brazil). Due to the perfect fit of tooth fragments and prior excess removal, minimal occlusion adjustment was needed.

### 2.7. Finishing and Polishing

Polishing provides a smooth surface of the teeth, thus, reducing the accumulation of dental plaque. While it is important, polishing removes the fluorine-rich enamel layer and should, therefore, be carried out selectively [78].

The restored teeth were first finished using fine and extra-fine diamond burs (2135F and 2135FF, respectively (KG, Sorensen, Cotia, SP, Brazil)), and finally polished using either a Soflex discs coarse, medium, fine, and super-fine grit Sof-Lex disk (3M ESPE, St. Paul, MN, USA) in a slow-speed hand piece for 30 s each.

### 2.8. Follow-Up Visits and Prognosis

The follow-up visits after trauma injury are of paramount importance and, therefore, mandatory. The control should consist of an interview, radiological examination, and pulp sensitivity tests. It enables the early detection and implementation of appropriate treatment to avoid long-term complications. The most common post-traumatic problems include pulp infection and necrosis, pulp canal obliteration (PCO) or root resorptions.

In the case of the complicated crown fractures, the recommended follow-up visits are as follows: after 14 days, 6–8 weeks, 3 and 6 months, and one year after the injury. In the case of the presented cases, the check-ups were carried out in accordance with the recommended scheme, and the positive results obtained after one year by interview, radiological examination, and pulp sensitivity cold/hot test indicate the success of the treatment.

## 3. Prognosis and Future Perspectives

If the pulp is exposed, biological treatment procedures should be performed. It is of paramount importance in the case of patients with open apices, as the preservation of the vital pulp ensures further physiological root formation. The prognosis of direct pulp capping with use of CH shows a success rate of 54–90% [25,79]. In addition, partial pulpotomy with that material may present a greater success rate (86–100%) [14,79,80]. The use of hydraulic calcium-silicate-based cements might contribute to the better prognosis of VPT than application of CH [81]. Moreover, total pulpotomy using CSMs has a success rate of 74–100% after 1 to 5 years follow-up [25,82,83]. Currently, there are reports presenting the possibility of including irreversibly damaged pulp in the indications for VPT [84,85].

The reported success rate of partial pulpotomy in permanent dentition with complicated crown fractures ranges from 87.5% to 100% [12,53]. The initial condition of the pulp, the absence of its damage, or the presence of any other trauma, which may affect blood supply, play a key role in the long-term treatment success [53].

The employment of adhesive techniques increases the success rate of the reattachment procedure by up to 84–93% [57,59]. Cases of successful treatment after 5 or even 9year follow-ups are reported [86,87]. The pulp treatment is not a factor that impaired the stability of the reattached fragments. When using the conventional etch and rinse technique, the bonding performance of the restoration mainly depends on the micromechanical retention between the composite resin and the etched enamel, as well as the hybrid layer. Therefore, the success rate is not affected by the pulp treatment [68]. Thus, the reattachment technique can be used both in uncomplicated and complicated crown fractures. Adhesive reattachment combined with vital pulp therapy procedures is a good first-choice treatment option in cases of complicated crown fractures.

Moreover, the use of toothpaste containing biomimetic hydroxyapatite for home management after reconstruction can reduce discoloration and hypersensitivity more effectively than conventional fluoride toothpaste [88,89].

## Figures and Tables

**Table 1:** Treatment options of crown fractures with pulpal exposure.

Vital Pulp Therapy (VPT) Intervention	Description of the Method	Indication
Direct pulp capping (DPC)	(1) Placement of protective pulp capping material directly over the exposure	A recent and pinpoint-sized exposed vital pulp
Partial pulpotomy (PP)	(1) Partial removal of the coronal pulp; (2) Hemostasis; (3) Placement of a pulp capping material.	Pulp exposure treated within 14 days after trauma, caries-free, open apex or thin dentinal walls, and vital and asymptomatic pulp
Full (complete) pulpotomy (CP)	(1) Removal of the entire coronal pulp to the level of canal orifices; (2) Hemostasis; (3) Placement of a pulp capping material.	More than 2 week lapse between trauma and treatment, extensive pulp exposure

## Data Availability

Not applicable.
